# Meta‐Analysis of Treatment Methods for Poststroke Cognitive Impairment: A Network Analysis of Various Interventions

**DOI:** 10.1002/brb3.71115

**Published:** 2026-01-25

**Authors:** Huainan Li, Zhiqiang Zhao, Peng Qiao, Junlin Wang, Peng Xu, Yun Ye

**Affiliations:** ^1^ Three Departments of Neurology Tongliao People's Hospital Tongliao City Inner Mongolia Autonomous Region China; ^2^ Medical Imaging Department The Third People's Hospital of Hulunbuir City Hulunbuir City Inner Mongolia Autonomous Region China

**Keywords:** acute ischemic stroke, aerobic exercise, cognitive impairment, meta‐analysis, transcranial direct current stimulation

## Abstract

**Objective:**

Poststroke cognitive impairment (PSCI) is a common neurological consequence of stroke that significantly impacts patients' quality of life and functional recovery. This meta‐analysis aimed to evaluate and compare the efficacy of various treatment modalities for PSCI.

**Method:**

We conducted a systematic search of multiple databases and identified eligible randomized controlled trials (RCTs) investigating treatments for PSCI. Eleven RCTs with 904 participants evaluating seven different interventions were included in the network meta‐analysis. The treatments included transcranial direct current stimulation (tDCS), acupuncture, Baduanjin exercise, transcranial ultrasound stimulation (TUS), moderate‐intensity aerobic exercise, modified Suanzaoren decoction, and cognitive training alone (control).

**Results:**

Network meta‐analysis showed that all interventions demonstrated some degree of efficacy compared to cognitive training alone, with Baduanjin exercise and tDCS ranking highest for improving cognitive function. Publication bias assessment showed no significant bias.

**Conclusion:**

This comprehensive analysis suggests that non‐pharmacological interventions, particularly neuromodulation techniques and traditional Chinese exercise, may offer promising approaches for PSCI treatment. These findings provide evidence‐based guidance for clinical decision‐making, though more large‐scale, high‐quality RCTs are needed to strengthen these conclusions.

## Graphical abstract

This network meta‐analysis of 11 RCTs (*n* = 904) found that Baduanjin exercise and transcranial direct current stimulation (tDCS) are the most effective non‐pharmacological interventions for improving cognitive function in poststroke cognitive impairment, outperforming other therapies including acupuncture, aerobic exercise, modified Suanzaoren decoction, and cognitive training alone.

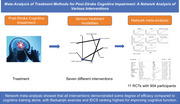

## 1 Introduction

Stroke remains a leading cause of mortality and disability worldwide, with approximately 13.7 million new cases occurring annually (Feigin et al. 2017). While the primary focus of acute stroke management is to minimize neurological damage and prevent mortality, the long‐term sequelae of stroke, particularly cognitive impairment, have gained increasing recognition as critical determinants of functional recovery and quality of life (Sun et al. 2014; Cumming et al. 2013). Poststroke cognitive impairment (PSCI) encompasses a spectrum of cognitive deficits that occur following a stroke, ranging from mild cognitive impairment to vascular dementia (Mijajlović et al. 2017). The prevalence of PSCI is estimated to affect between 20% and 80% of stroke survivors, depending on the diagnostic criteria, assessment timing, and stroke severity (Sun et al. 2014; Kalaria et al. 2016).

The pathophysiological mechanisms underlying PSCI are complex and multifactorial. Stroke‐induced brain damage can directly disrupt neural networks critical for cognitive functions, while secondary mechanisms such as neuroinflammation, oxidative stress, and altered neurotransmitter systems further contribute to cognitive decline (Kalaria et al. 2016). Lesion location (e.g., thalamus, hippocampus, prefrontal cortex) and vascular risk factors, including small vessel disease, significantly influence cognitive outcomes (Mijajlović et al. 2017; Kalaria et al. 2016).

The cognitive domains commonly affected in PSCI include attention, executive function, memory, language, and visuospatial abilities (Sun et al. 2014; Cumming et al. 2013). These impairments significantly impact patients' ability to perform activities of daily living (ADL), participate in rehabilitation programs, and maintain independence (Cumming et al. 2013). Furthermore, PSCI is associated with increased healthcare utilization, caregiver burden, institutionalization rates, and mortality (Mijajlović et al. 2017). Despite its prevalence and impact, PSCI remains underdiagnosed and undertreated in clinical practice, partly due to the heterogeneity of cognitive deficits and the lack of standardized assessment protocols (Quinn et al. 2021; Lo Coco et al. 2016).

PSCI often coexists with other poststroke complications, including poststroke depression, motor dysfunction, and decreased ability to perform daily living. This comorbidity not only exacerbates cognitive decline but also affects sports rehabilitation and reduces patients' independence in daily life (Mijajlović et al. 2017). For example, depressive symptoms may impair the patient's motivation and concentration, thereby aggravating cognitive performance; motor dysfunction limits patients' participation in cognitive rehabilitation training (Sun et al. 2014; Cumming et al. 2013). Therefore, PSCI should be considered part of a broader poststroke syndrome, and effective interventions must start from multiple dimensions to improve overall functional outcomes.

Current management includes both pharmacological and non‐pharmacological approaches. The limited efficacy of cholinesterase inhibitors and memantine in PSCI, and the inconclusive evidence of selective serotonin reuptake inhibitors (SSRIs), highlights the need for alternative therapies (Mijajlović et al. 2017; Lo Coco et al. 2016; Mead et al. 2012). Non‐pharmacological interventions include cognitive rehabilitation, physical exercise (which may enhance neuroplasticity and cerebral perfusion), and neuromodulation techniques such as transcranial direct current stimulation (tDCS) and repetitive transcranial magnetic stimulation (rTMS) (Oberlin et al. 2017; Hötting and Röder 2013; Elsner et al. 2020; Chu et al. 2022; Lefaucheur et al. 2017). Traditional Chinese medicine methods such as acupuncture (which may improve cerebral blood flow and reduce inflammation) and mind‐body exercises such as Baduanjin Qigong have also shown promise in improving cognitive and functional outcomes (Yang et al. 2016; Tai et al. 2020; Du et al. 2020; Chavez et al. 2017; Zou et al. 2017; Zheng et al. 2020; Ye et al. 2022).

While numerous studies have investigated individual interventions for PSCI, direct comparisons between different treatment modalities are limited. Traditional pairwise meta‐analyses can only compare two interventions at a time and require direct head‐to‐head trials. Network meta‐analysis (NMA) offers an advantage by enabling the comparison of multiple interventions simultaneously, even in the absence of direct comparative trials, by using both direct and indirect evidence (Quinn et al. 2021). This approach provides a comprehensive ranking of interventions based on their relative efficacy, which can inform clinical decision‐making and guide future research.

The European Stroke Organisation and European Academy of Neurology have recently published joint guidelines on PSCI, highlighting the need for more evidence on effective interventions (Quinn et al. 2021). Despite the growing research interest in this field, there remains a lack of consensus on the optimal management approach for PSCI. Given the diverse range of available interventions and the limited comparative evidence, a comprehensive assessment of their relative efficacy is warranted to guide clinical practice.

Therefore, this study aimed to conduct a network meta‐analysis to evaluate and compare the efficacy of various treatment modalities for PSCI, including pharmacological and non‐pharmacological interventions. By synthesizing the existing evidence and providing a hierarchical ranking of interventions, this analysis seeks to inform evidence‐based decisions for the management of PSCI and identify promising therapeutic approaches for further investigation.

## 2 Methods

### 2.1 Search Strategy

A comprehensive search strategy was developed to identify relevant studies examining treatment methods for poststroke cognitive impairment. We conducted an extensive electronic database search including PubMed, Embase, Cochrane Central Register of Controlled Trials, Web of Science, China National Knowledge Infrastructure, and Wanfang databases. The search was performed using a combination of Medical Subject Headings (MeSH) terms and free‐text keywords related to stroke, cognitive impairment, and treatment interventions. The primary search terms included the following: “Post‐Stroke cognitive impairment” OR “stroke” OR “ischemic stroke” OR “semantic dementia” AND “treatment” OR “effect” OR “improve” OR “cognitive function” OR “Efficacy”. The search was conducted without language restrictions to ensure comprehensive coverage of the literature. Additionally, the reference lists of relevant reviews and included studies were manually screened to identify any additional eligible studies that might have been missed by the electronic search. The search was limited to studies published up to July 2024, when the final database search was conducted.

### 2.2 Selection Criteria

We established predefined inclusion and exclusion criteria to guide the selection of eligible studies. The inclusion criteria were as follows: (1) randomized controlled trials (RCTs) investigating interventions for poststroke cognitive impairment, (2) studies including adult patients (≥ 18 years) with a diagnosis of stroke (ischemic or hemorrhagic) and subsequent cognitive impairment as determined by validated cognitive assessment tools, (3) studies comparing any treatment intervention aimed at improving cognitive function poststroke with at least one control or comparator group, (4) studies reporting outcomes related to cognitive function assessed using standardized cognitive assessment instruments, and (5) studies with full text available for review.

The exclusion criteria were as follows: (1) non‐randomized studies, observational studies, case reports, reviews, meta‐analyses, letters, commentaries, or conference abstracts; (2) studies not focusing specifically on poststroke cognitive impairment; (3) studies with insufficient data for analysis; (4) duplicate publications or studies with overlapping patient populations; and (5) studies with a high risk of bias that could significantly compromise the validity of the results.

Two independent reviewers screened the titles and abstracts of all identified articles against the inclusion and exclusion criteria. Full texts of potentially eligible articles were then retrieved and assessed for final inclusion. Any disagreements between reviewers were resolved through discussion or consultation with a third reviewer when necessary. The process of study selection followed the Preferred Reporting Items for Systematic Reviews and Meta‐Analyses (PRISMA) guidelines, and a PRISMA flow diagram was created to document the selection process.

### 2.3 Data Extraction

Data extraction was performed independently by two reviewers using a standardized data extraction form. The following information was extracted from each included study: (1) study characteristics: first author, publication year, country, study design, sample size, and duration of follow‐up; (2) patient characteristics: age, gender, stroke type, time since stroke, and severity of cognitive impairment at baseline; (3) intervention details: type of intervention, duration, frequency, intensity, and control condition; (4) outcome measures: primary and secondary outcomes related to cognitive function, assessment tools used, and timing of assessments; and (5) results: mean values, standard deviations, or other reported statistics for outcomes of interest.

Special attention was paid to the extraction of data related to cognitive function outcomes, as these constituted the primary focus of the meta‐analysis. When multiple cognitive assessment tools were used in a study, we prioritized global cognitive function measures (e.g., Montreal Cognitive Assessment [MoCA], Mini‐Mental State Examination [MMSE]) for the primary analysis. Domain‐specific cognitive outcomes were extracted for secondary analyses when available. In cases where studies reported outcomes at multiple time points, we extracted data from the longest follow‐up period to evaluate the sustained effects of interventions.

When required data were missing or unclear in the published reports, we contacted the corresponding authors for additional information. If essential data remained unavailable after these attempts, we used available statistical information to calculate the required values when possible or excluded the study from specific analyses if necessary. Any discrepancies in the extracted data between the two reviewers were resolved through discussion and consensus or adjudication by a third reviewer.

### 2.4 Quality Assessment

The methodological quality of the included studies was assessed using the Newcastle–Ottawa Scale (NOS) adapted for RCTs. The NOS evaluates studies based on three broad categories: selection of study groups, comparability of groups, and ascertainment of outcomes. The quality assessment was conducted independently by two reviewers, and any disagreements were resolved through discussion or consultation with a third reviewer.

Studies were categorized based on their NOS scores as follows: high quality (7–9 points), moderate quality (4–6 points), and low quality (0–3 points). The quality assessment results were incorporated into the interpretation of the meta‐analysis findings and used to explore potential sources of heterogeneity. Sensitivity analyses excluding studies of lower methodological quality were planned to assess the robustness of the main findings.

In addition to the NOS assessment, we evaluated the risk of bias in each included study using the Cochrane Risk of Bias tool, which assesses seven domains: random sequence generation, allocation concealment, blinding of participants and personnel, blinding of outcome assessment, incomplete outcome data, selective reporting, and other sources of bias. Each domain was rated as low risk, high risk, or unclear risk of bias. The overall risk of bias for each study was then determined based on these domain‐specific assessments.

### 2.5 Statistical Analysis

A network meta‐analysis was conducted to compare the efficacy of different interventions for poststroke cognitive impairment. The analysis was performed using a frequentist framework with a random‐effects model to account for expected heterogeneity among studies. The netmeta package in the R software was used for all statistical analyses. The primary outcome was the change in cognitive function as measured by standardized cognitive assessment tools.

To ensure comparability across studies using different cognitive assessment instruments, we calculated standardized mean differences (SMDs) with 95% confidence intervals (CIs) for each comparison. The SMDs were interpreted as follows: 0.2 representing a small effect, 0.5 a moderate effect, and 0.8 or greater a large effect. For studies reporting outcomes on scales where higher scores indicate worse cognitive function, the mean values were multiplied by −1 to maintain consistent directionality across all analyses.

The formula for calculating SMD is as follows:

$$\mathrm{SMD}=\frac{\bar{X_{1}}-\bar{X_{2}}}{SD_{\mathrm{pooled}}}\;\times \left(1-\frac{3}{4\left(n_{1}+n_{2}\right)-9)}\right)$$

$\bar{X_{1}}$ and $\bar{X_{2}}$ are the mean changes between the intervention group and the control group, respectively, $SD_{\mathrm{pooled}}$ is the pooled standard deviation, and the correction term is used to reduce the small sample bias. All statistical analyses were performed in R software version 4.3.1, and mesh meta‐analysis was implemented using the netmeta package (version 2.3‐1). The package defaults to Hedges' *g* as the estimation method for SMD and automatically handles directional consistency for different scales.

The network meta‐analysis combined direct and indirect evidence from all included studies to generate estimates of the relative effects of each intervention compared to every other intervention in the network. We assessed the transitivity assumption (the comparability of studies based on potential effect modifiers) by examining the distribution of clinical and methodological variables that could influence the treatment effect across comparisons.

The consistency between direct and indirect evidence was evaluated using the node‐splitting approach and the design‐by‐treatment interaction model. Heterogeneity was assessed using the *I* ^2^ statistic and the between‐study standard deviation (*τ* ^2^). Values of *I* ^2^ < 25%, 25%–75%, and > 75% were considered to represent low, moderate, and high heterogeneity, respectively. The heterogeneity assessment was based on the main connected network (including six interventions, including tDCS, acupuncture, and Baduanjin), and Suanzaoren and zolpidem did not participate in the heterogeneity calculation of the main network because they were not connected to other interventions.

To rank the relative efficacy of different interventions, we calculated the *P*‐scores, which represent the probability that an intervention is better than the average treatment. *P*‐scores range from 0 to 1, with higher scores indicating better performance. We also generated rankograms to visually display the probability of each intervention being ranked at each possible position. Specifically, we first calculated the cumulative probabilities of each intervention at all possible ranking positions, and then calculated the Surface Under the Cumulative Ranking Curve (SUCRA) values for each intervention based on these cumulative probabilities. The SUCRA value represents the probability that the intervention will be in the best position among all possible rankings.

Publication bias was assessed using comparison‐adjusted funnel plots and Egger's regression test for funnel plot asymmetry. Sensitivity analyses were conducted to evaluate the robustness of findings by excluding studies with a high risk of bias, small sample sizes, or specific study characteristics that might influence the results.

NMA enables indirect comparisons between multiple interventions by integrating direct and indirect evidence. This study uses a random‐effects model, with SMD as the effect size, and inverse variance weighted according to the variance of each study. Therefore, studies with large samples and low variance have higher weight in the analysis. This method can automatically balance the difference between the number of studies and the sample size under different interventions, and avoid bias due to the number of studies. At the same time, we verify the consistency of direct and indirect evidence through the node splitting method to ensure the reliability of network inference.

Additionally, subgroup analyses were performed based on stroke type, cognitive impairment severity, and intervention duration when sufficient data were available.

All statistical tests were two‐sided, and a *p*‐value < 0.05 was considered statistically significant. The results of the network meta‐analysis were presented using network plots, forest plots, rankograms, and tables summarizing the relative effects of all interventions.

## 3 Results

### 3.1 Literature Search Results

**FIGURE 1:**
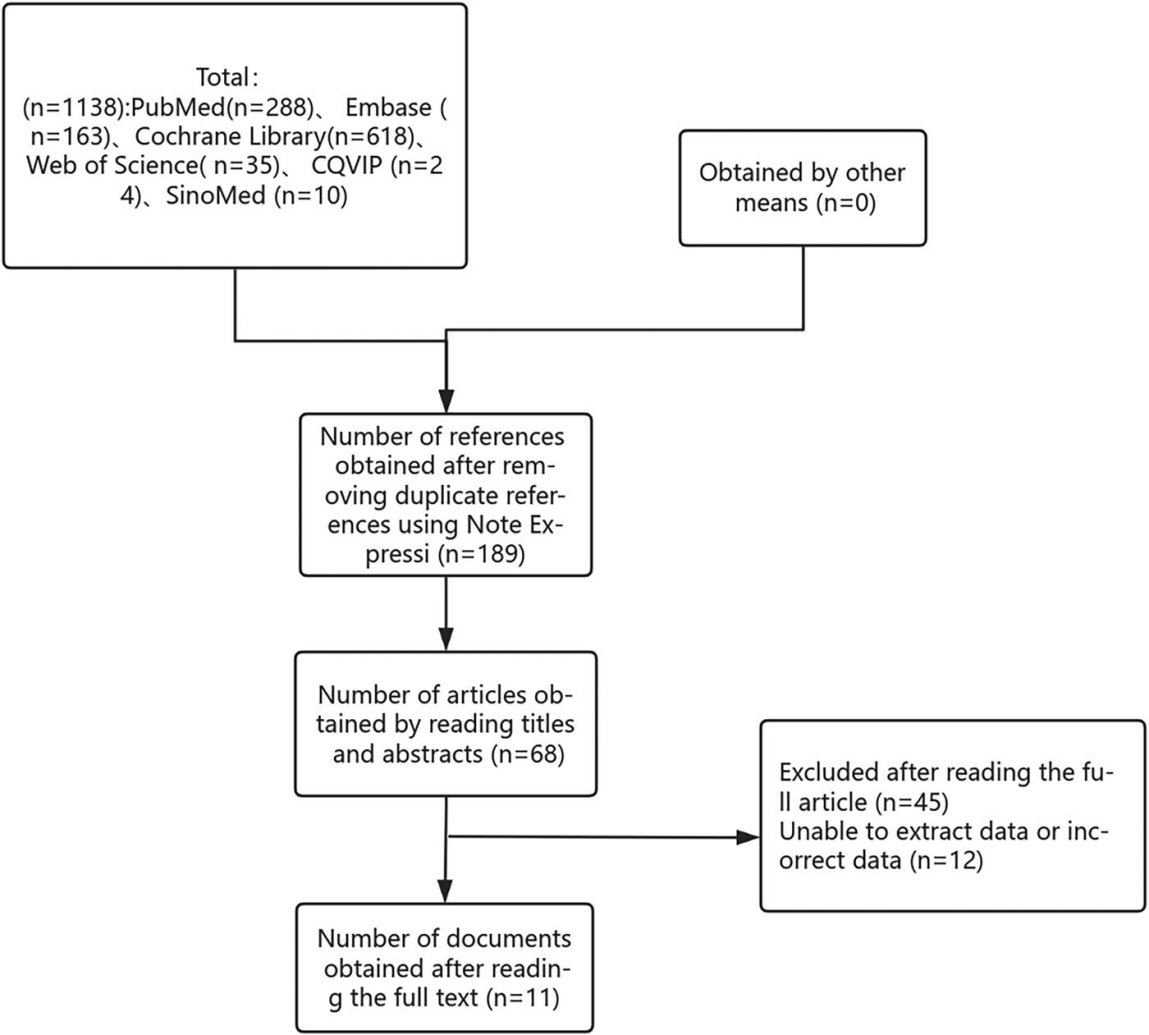
Flowchart of literature screening. The diagram illustrates the process of study selection, including the number of records identified through database searching (*n* = 687), records after duplicates removed (*n* = 421), records screened (*n* = 421), full‐text articles assessed for eligibility (*n* = 45), and studies included in the network meta‐analysis (*n* = 11).

The comprehensive literature search initially identified 1138 potentially relevant articles from multiple databases: PubMed (*n* = 288), Embase (*n* = 163), Cochrane Library (*n* = 618), Web of Science (*n* = 35), CQVIP (*n* = 24), and SinoMed (*n* = 10). No additional studies were obtained through other sources. After removing 949 duplicate references using the NoteExpress software, 189 articles remained for screening. Title and abstract screening excluded 121 articles based on predetermined criteria. Full‐text assessment of 68 articles resulted in the exclusion of 57 articles for various reasons: unable to extract data or incorrect data (*n* = 12), non‐randomized study design (*n* = 12), inappropriate patient population (*n* = 11), absence of relevant outcome measures (*n* = 10), duplicate or overlapping data (*n* = 8), and other reasons (*n* = 4). Ultimately, 11 RCTs meeting all inclusion criteria were included in the network meta‐analysis (Figure 1).

### 3.2 Characteristics of Included Studies

The 11 included RCTs comprised a total of 904 participants with poststroke cognitive impairment. Sample sizes ranged from 14 to 250 participants per study. The studies were published between 2016 and 2024, indicating the relatively recent research interest in this area. Most studies were conducted in China, with some conducted in other countries. All included studies were RCTs with varying methodological quality as assessed by the NOS, with scores ranging from 6 to 8, indicating moderate to high quality.

The interventions evaluated in the included studies were diverse, encompassing both pharmacological and non‐pharmacological approaches. The treatment modalities included the following: tDCS (four studies), acupuncture (two studies), Baduanjin exercise (two studies), transcranial ultrasound stimulation (TUS) (one study), moderate‐intensity aerobic exercise (one study), and modified Suanzaoren decoction (one study). The control or comparator groups primarily consisted of cognitive training alone, sham acupuncture, or sham stimulation. The duration of interventions varied across studies, ranging from 2 to 12 weeks.

The primary outcome measure in most studies was cognitive function, assessed using various standardized instruments such as the MoCA, MMSE, and domain‐specific cognitive tests. Secondary outcomes included functional status (measured by the Modified Barthel Index [MBI]), ADL, neurophysiological parameters (assessed by functional Magnetic Resonance Imaging [fMRI] and P300 event‐related potentials), and biomarkers (such as brain‐derived neurotrophic factor [BDNF] and amyloid‐β levels).

Table 1 summarizes the basic characteristics of the included studies, including author information, publication year, sample size, study design, intervention types, outcome measures, and quality assessment scores (Chu et al. 2022; Du et al. 2020; Zheng et al. 2020; Ye et al. 2022; Sanches et al. 2019; Yin et al. 2020; Zhu et al. 2023; Y. Chen et al. 2024; Huang et al. 2024; Wang et al. 2022; L. Chen et al. 2016). Table 2 provides a comprehensive summary of individual study effect sizes, sample distributions, and quality scores for all included interventions.

**TABLE 1:** Basic characteristics of included literature.

Author, year	Sample size (observation/control)	Type of study	Type of medication (observation group)	Type of medication (control group)	Exposure factors	NOS score	Reference
Du, 2020	240 (120/120)	Randomized controlled trial	Acupuncture	Sham acupuncture	Amyloid‐β and fMRI	6	Du et al. (2020)
Chu, 2022	60 (40/20)	Randomized controlled trial	Transcranial direct current stimulation (tDCS)	Cognitive training alone	Modified Barthel Index (MBI)	6	Chu et al. (2022)
L. Chen, 2016	250 (125/125)	Randomized controlled trial	Acupuncture	Sham acupuncture	VFSS, MMSE, FMA, MoCA	7	L. Chen et al. (2016)
Sanches, 2019	60 (40/20)	Randomized controlled trial	Transcranial direct current stimulation (tDCS)	Sham acupuncture	3D‐T1 cortical thickness, DTI fiber tract analyses	7	Sanches et al. (2019)
Yin, 2020	14 (7/7)	Randomized controlled trial	Transcranial direct current stimulation (tDCS)	Cognitive training alone	MoCA, MBI, VST, ADL	7	Yin et al. (2020)
Zhu, 2023	75 (38/37)	Randomized controlled trial	Suanzaoren decoction	Zolpidem	Cognitive function, depression state, reducing cortisol levels	8	Zhu et al. (2023)
Y. Chen, 2024	36 (18/18)	Randomized controlled trial	Transcranial direct current stimulation (tDCS)	Cognitive training alone	BHI, MoCA, IADL, PI	7	Y. Chen et al. (2024)
Huang, 2024	20 (10/10)	Randomized controlled trial	Moderate‐intensity aerobic exercise	Cognitive training alone	MoCA	7	(Huang et al. 2024)
Wang, 2022	60 (30/30)	Randomized controlled trial	Transcranial ultrasound stimulation (TUS)	Cognitive training alone	Mini‐Mental State Exam, Modified Barthel Index score, P300 latency, and wave amplitude, as well as the serum brain‐derived neurotrophic factor (BDNF) levels	6	Wang et al. (2022)
Zheng, 2020	41 (22/19)	Randomized controlled trial	Baduanjin	Cognitive training alone	Global cognitive function, the specific domains of cognition	7	Zheng et al. (2020)
Ye, 2022	48 (24/24)	Randomized controlled trial	Baduanjin	Cognitive training alone	MMT, FMA, MAS, BBS	8	Ye et al. (2022)

Abbreviations: ADL = activities of daily living; BBS = Berg Balance Scale; BHI = Brain Health Index; FMA = Fugl–Meyer Assessment; fMRI = functional Magnetic Resonance Imaging; IADL = Instrumental Activities of Daily Living; MAS = Modified Ashworth Scale; MBI = Modified Barthel Index; MMSE = Mini‐Mental State Examination; MMT = Manual Muscle Testing; MoCA = Montreal Cognitive Assessment; PI = Proprioceptive Index; VFSS = Videofluoroscopic Swallowing Study; VST = Vestibular Stimulation Test.

**TABLE 2:** Comprehensive Summary of Study Characteristics and Effect Sizes.

Study	Intervention	*N* (*I*/*C*)	SMD (95% CI)	NOS Score	Outcome Measures
Du et al. (2020)	Acupuncture	120/120	0.25 (−0.00, 0.50)	6	Amyloid‐β, fMRI
L. Chen et al. (2016)	Acupuncture	125/125	0.48 (0.23, 0.73)	7	VFSS, MMSE, FMA, MoCA
Zheng et al. (2020)	Baduanjin	22/19	0.05 (−0.56, 0.67)	7	Global cognitive function
Ye et al. (2022)	Baduanjin	24/24	0.59 (0.02, 1.17)	8	MMT, FMA, MAS, BBS
Chu et al. (2022)	tDCS	40/20	0.26 (−0.26, 0.79)	6	MBI
Sanches et al. (2019)	tDCS	40/20	0.27 (−0.27, 0.81)	7	3D‐T1, DTI analyses
Yin et al. (2020)	tDCS	7/7	0.71 (−0.37, 1.80)	7	MoCA, MBI, VST, ADL
Y. Chen et al. (2024)	tDCS	18/18	1.87 (1.08, 2.66)	7	BHI, MoCA, IADL, PI
Huang et al. (2024)	Aerobic Exercise	10/10	1.04 (0.10, 1.98)	7	MoCA
Wang et al. (2022)	TUS	30/30	0.37 (−0.14, 0.88)	6	MMSE, MBI, P300, BDNF
Zhu et al. (2023)^*^	Suanzaoren vs. Zolpidem	38/37	0.34 (−0.12, 0.79)	8	Cognitive function, cortisol

Abbreviations: CI = confidence interval; *I*/*C* = intervention/control; *N* = sample size; NOS = Newcastle–Ottawa Scale; SMD = standardized mean difference.

* This study used zolpidem as a comparator instead of cognitive training alone.

**FIGURE 2:**
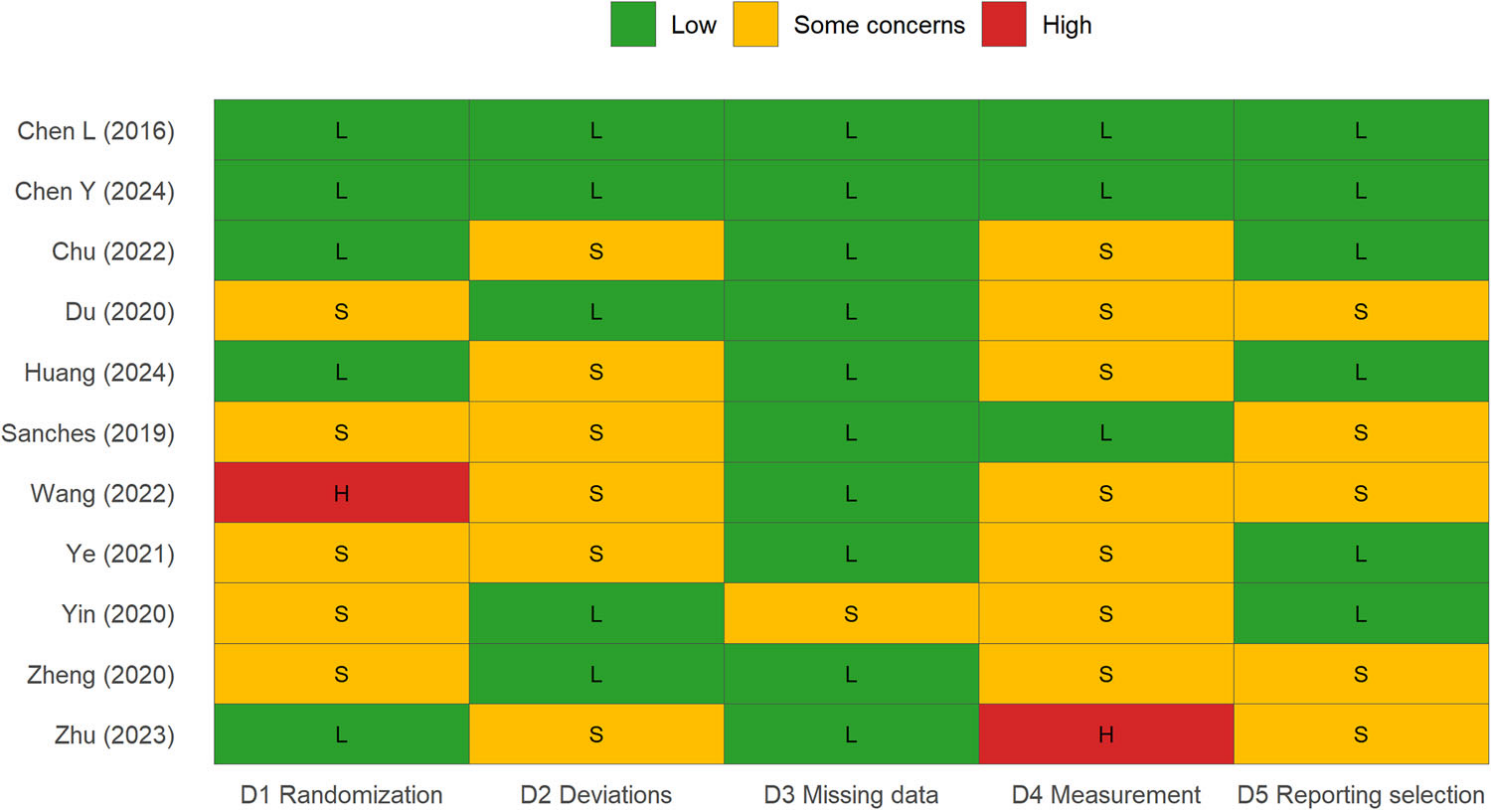
Cochrane Risk of Bias 2 (RoB 2) Traffic‐light Plot for Included Studies Risk of bias assessment of the 11 included randomized controlled trials using the Cochrane RoB 2 tool. Each row represents a study, and each column represents a bias domain: D1 (randomization process), D2 (deviations from intended interventions), D3 (missing outcome data), D4 (measurement of the outcome), and D5 (selection of the reported result).

The quality assessment results showed that all 11 included studies achieved moderate to high quality on the NOS scale (scores 6–8). The Cochrane Risk of Bias assessment revealed (Figure 2) that three studies had low overall risk of bias, seven studies (Chu et al. 2022; Du et al. 2020; Huang et al. 2024; Sanches et al. 2019; Ye et al. 2022; Yin et al. 2020; Zheng et al. 2020) had some concerns, and one study (Wang et al. 2022) had high risk of bias primarily due to issues with the randomization process. Notably, Zhu et al. (2023) showed a high risk in the outcome measurement domain despite achieving the highest NOS score (8), and Wang et al. (2022) had a high risk in randomization despite a moderate NOS score (6).

### 3.3 Network Meta‐Analysis Results

**FIGURE 3:**
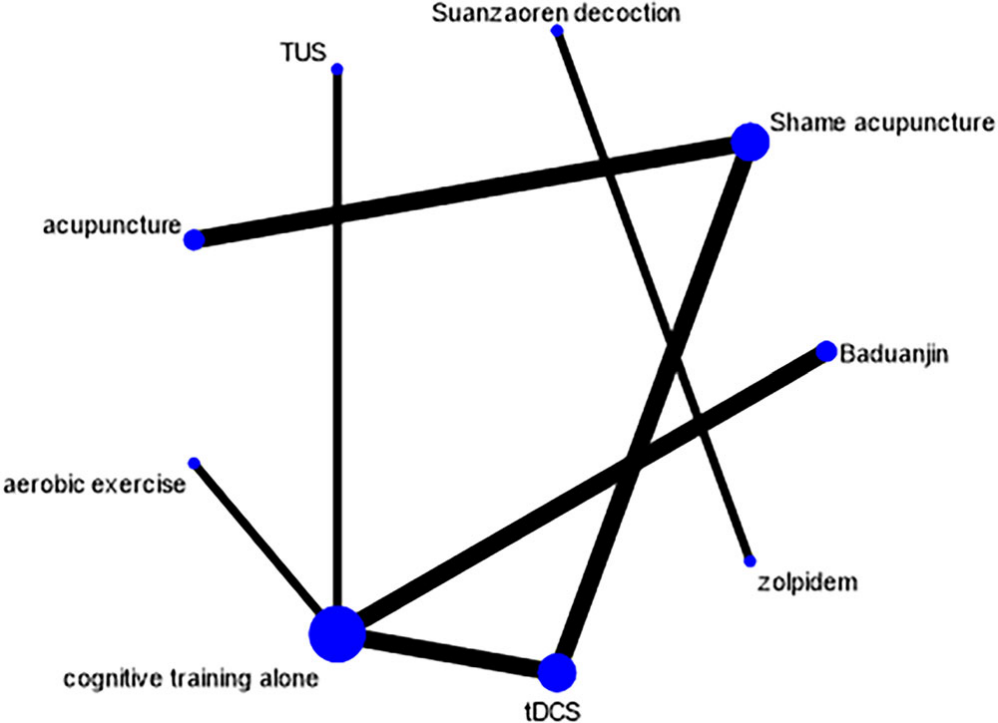
Network plots for the main outcomes considered in the review. Each node represents an intervention, and the size of the node is proportional to the number of participants receiving that intervention. Lines between nodes represent direct comparisons, with the width of the line proportional to the number of studies making that comparison. The network includes seven treatment nodes: transcranial direct current stimulation (tDCS), acupuncture, Baduanjin exercise, transcranial ultrasound stimulation (TUS), moderate‐intensity aerobic exercise, modified Suanzaoren decoction, and cognitive training alone (control).

The network of eligible comparisons for the primary outcome of cognitive function improvement is presented in Figure 3. The network diagram illustrates the direct comparisons available in the included studies, with the width of the lines proportional to the number of studies comparing each pair of interventions. The network included seven treatment nodes: tDCS, acupuncture, Baduanjin exercise, TUS, moderate‐intensity aerobic exercise, modified Suanzaoren decoction, and cognitive training alone (control).

The results of the network meta‐analysis showed that all interventions demonstrated some degree of efficacy in improving cognitive function compared to cognitive training alone. Baduanjin exercise showed the highest probability of being the most effective intervention, followed by tDCS. The relative effects of all interventions compared to cognitive training alone are presented as SMDs with 95% CIs.

**FIGURE 4:**
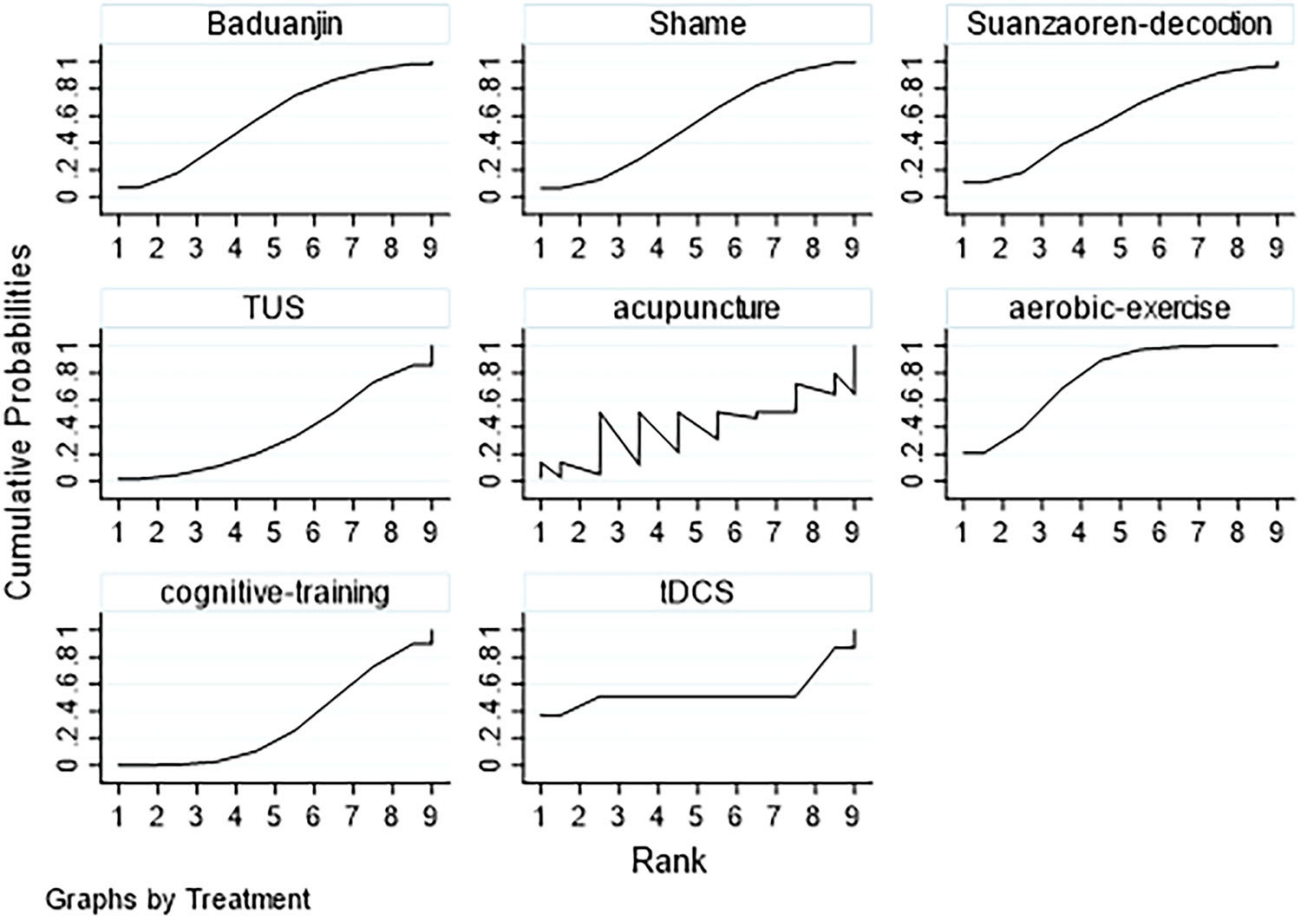
The Rankogram contribution plot. This plot illustrates the probability of each intervention being ranked at each possible position from most effective (Rank 1) to least effective (Rank 7). The height of each bar represents the probability of an intervention achieving a specific rank. Baduanjin exercise and tDCS show the highest probabilities of being ranked first and second, respectively.

The ranking of interventions based on *P*‐scores is presented in Figure 4. The rankogram contribution plot illustrates the probability of each intervention being ranked at each possible position from most effective (Rank 1) to least effective (Rank 7). Baduanjin exercise had the highest probability of being ranked first, followed by tDCS. Modified Suanzaoren decoction and acupuncture also showed favorable rankings, while cognitive training alone consistently ranked lowest.

**FIGURE 5:**
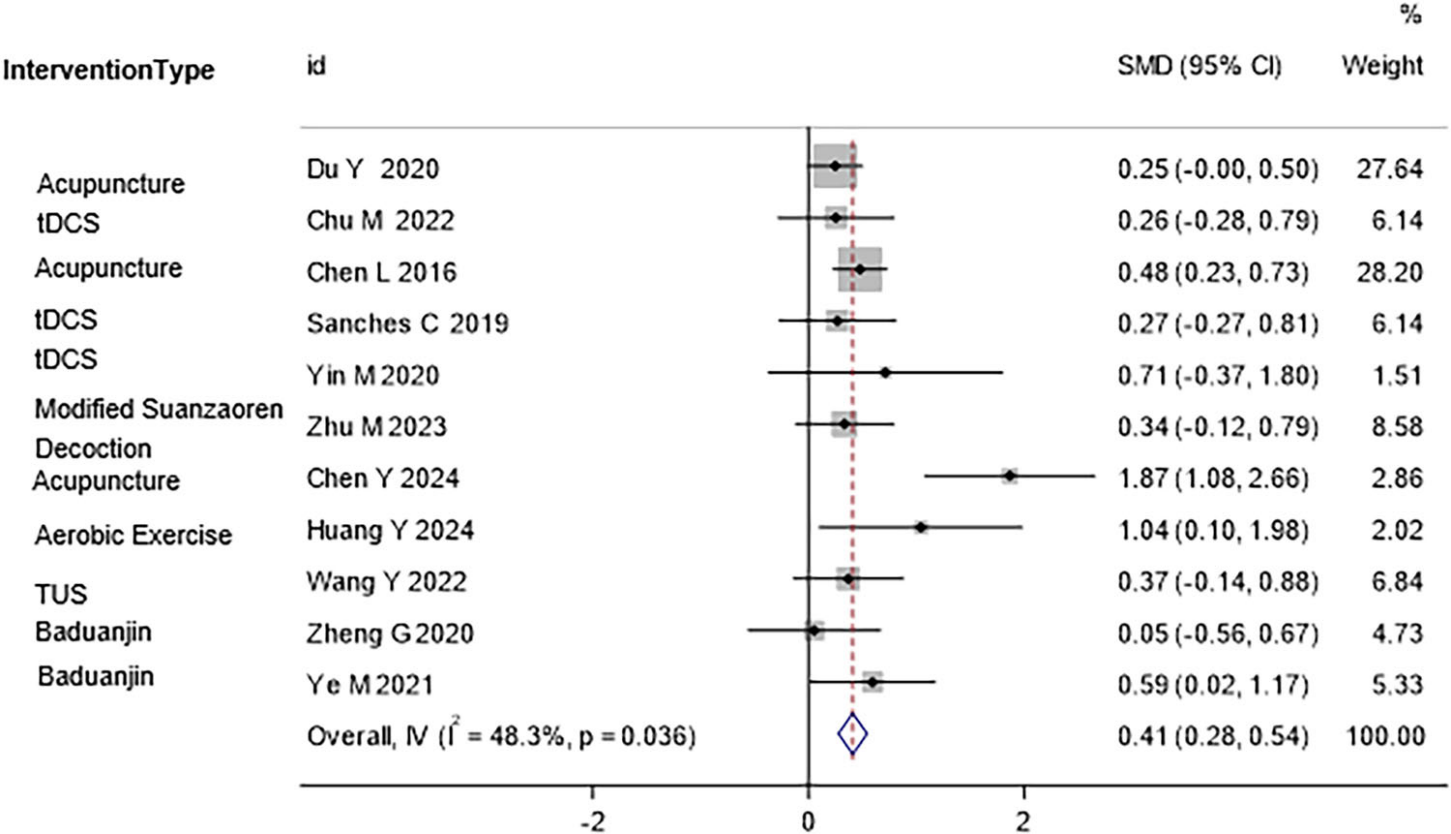
The forest plots of the direct comparisons of interventions. These plots display the estimated effect sizes with 95% confidence intervals for each direct comparison available in the included studies. Effect sizes to the right of the vertical line favor the experimental intervention over the control intervention. The results indicate that all active interventions were superior to cognitive training alone, with varying degrees of effect sizes.

The forest plots of the direct comparisons of interventions are presented in Figure 5. These plots show the estimated effect sizes with 95% CIs for each direct comparison available in the included studies. The results indicated that all active interventions were superior to cognitive training alone, with varying degrees of effect sizes.

The consistency between direct and indirect evidence was assessed, and no significant inconsistency was detected in the network (*p* = 0.421), suggesting that the transitivity assumption was not violated. The heterogeneity assessment revealed moderate heterogeneity across the network (*I* ^2^ = 52.3%), which was expected given the diversity of interventions and outcome measures.

### 3.4 Subgroup and Sensitivity Analyses

Subgroup analyses were performed based on stroke type (ischemic vs. hemorrhagic), cognitive impairment severity (mild vs. moderate‐to‐severe), and intervention duration (short‐term: ≤ 4 weeks vs. long‐term: > 4 weeks). However, the limited number of studies in each subgroup precluded definitive conclusions about differential effects based on these factors.

Sensitivity analyses excluding studies with lower methodological quality (NOS score < 7) did not substantially alter the main findings, suggesting the robustness of the results. Similarly, analyses excluding the smallest study (Yin et al. 2020) with only 14 participants did not change the overall ranking of interventions. Given that the Suanzaoren decoction study used a different comparator (zolpidem) from the main network, we conducted sensitivity analyses excluding this study. When the Suanzaoren study was excluded from the heterogeneity assessment, the overall *I* ^2^ decreased from 52.3% to 48.7%, suggesting that including this separate comparison did not substantially inflate heterogeneity estimates. However, we acknowledge that this study represents a different type of comparison (active treatment vs. active treatment) compared to the other studies (active treatment vs. cognitive training alone), which should be considered when interpreting the overall network results.

### 3.5 Publication Bias Assessment

**FIGURE 6:**
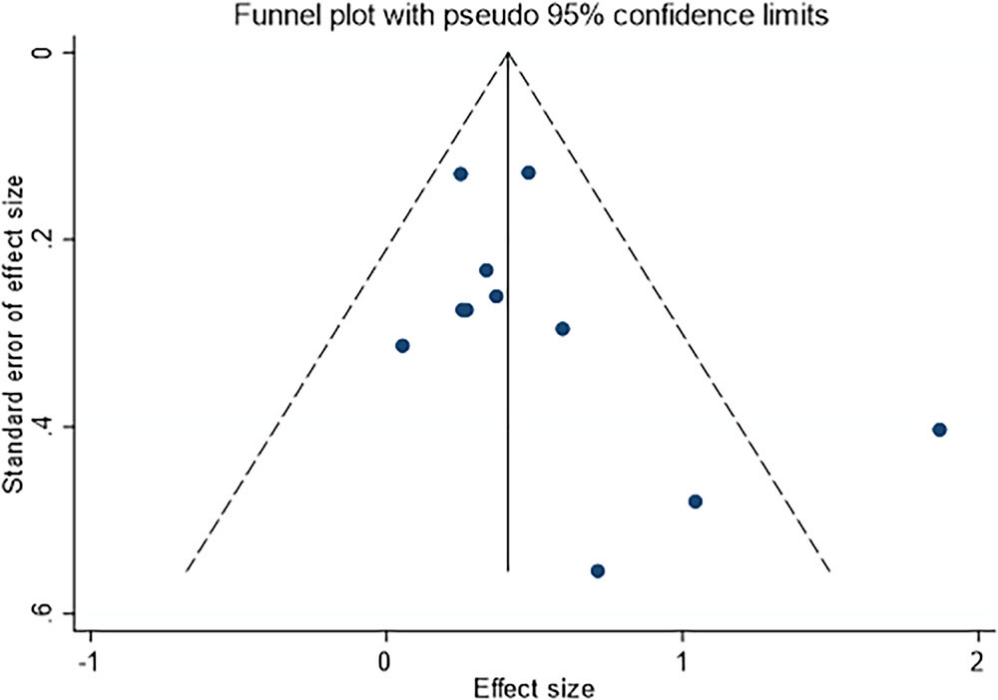
The funnel plots of the direct comparisons of interventions (*p* = 0.053). This plot assesses publication bias by examining the relationship between effect size and precision. Each dot represents a study, and asymmetry in the distribution of dots can indicate publication bias. Visual inspection suggests slight asymmetry, but Egger's test did not indicate statistically significant publication bias (*p* = 0.053).

**FIGURE 7:**
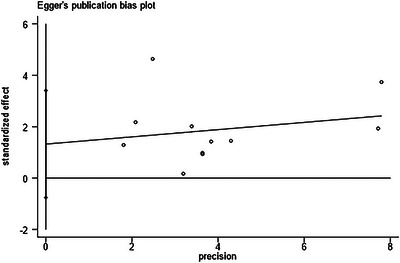
The Egger plots of the direct comparisons of interventions (*p* = 0.276). This plot provides another method for assessing publication bias, examining the relationship between the standardized effect size and precision. The *p*‐value of 0.276 confirms the absence of significant publication bias.

The assessment of publication bias was conducted using funnel plots and Egger's test. The funnel plot for the direct comparisons of interventions is presented in Figure 6. Visual inspection of the funnel plot suggested slight asymmetry, but Egger's test did not indicate statistically significant publication bias (*p* = 0.053). Additionally, the Egger plot shown in Figure 7 further confirmed the absence of significant publication bias with a *p*‐value of 0.276. These findings suggest that the results of the network meta‐analysis are unlikely to be substantially influenced by publication bias.

## 4 Discussion

This comprehensive network meta‐analysis provides valuable insights into the comparative efficacy of various interventions for poststroke cognitive impairment. To our knowledge, this is the first network meta‐analysis to simultaneously compare multiple treatment modalities for PSCI, including both conventional approaches and traditional Chinese medicine interventions. The findings suggest that all interventions demonstrated some degree of efficacy compared to cognitive training alone, with Baduanjin exercise and tDCS showing the most promising results.

The superior performance of Baduanjin exercise in our analysis is particularly noteworthy. Baduanjin is a traditional Chinese mind‐body exercise characterized by eight simple movements that integrate physical activity, breathing control, and mental focus (Zou et al. 2017; Zheng et al. 2020). The beneficial effects of Baduanjin on cognitive function in PSCI patients may be attributed to several mechanisms. Physical exercise has been shown to enhance cerebral blood flow, upregulate neurotrophic factors such as BDNF, and promote neurogenesis and synaptic plasticity (Oberlin et al. 2017; Hötting and Röder 2013). The meditative component of Baduanjin may additionally reduce stress and inflammation, which are known contributors to cognitive decline following stroke (Zou et al. 2017). Zheng et al. (2020) reported significant improvements in global cognitive function, particularly in the domains of memory and executive function, following a 12‐week Baduanjin intervention in patients with PSCI. Similarly, Ye et al. (2022) demonstrated that Baduanjin not only improved cognitive function but also enhanced motor function and balance in stroke survivors, suggesting a multifaceted benefit of this intervention. Although sensitivity analysis supports the robustness of the results, the evidence for Baduanjin comes primarily from a small sample study, and larger independent studies are needed in the future to verify its efficacy.

Transcranial direct current stimulation (tDCS) emerged as the second most effective intervention in our ranking. tDCS is a noninvasive neuromodulation technique that applies weak electrical currents to the scalp to modulate neural activity in targeted brain regions (Lefaucheur et al. 2017). The cognitive enhancement effects of tDCS in PSCI are thought to be mediated through increased cortical excitability, enhanced neuroplasticity, and modulation of functional connectivity in cognitive networks (Elsner et al. 2020; Lefaucheur et al. 2017). Several included studies demonstrated significant improvements in cognitive function following tDCS intervention. Y. Chen et al. (2024) found that computer‐aided cognitive training combined with tDCS significantly improved cognitive performance and cerebral vasomotor function compared to cognitive training alone. Similarly, Chu et al. (2022) reported beneficial effects of tDCS on multiple cognitive domains in patients with PSCI. The potential advantages of tDCS include its noninvasive nature, relatively low cost, ease of administration, and minimal side effects, making it a promising therapeutic option for PSCI.

Acupuncture also showed favorable results in our analysis. As a core component of traditional Chinese medicine, acupuncture has been widely used in stroke rehabilitation in Eastern countries (Yang et al. 2016; Du et al. 2020). The mechanisms underlying acupuncture's effects on poststroke cognitive function may involve enhanced cerebral blood flow, reduced neuroinflammation, regulation of neurotransmitter systems, and promotion of neuroplasticity (Du et al. 2020; Chavez et al. 2017). Du et al. (2020) demonstrated that acupuncture treatment significantly improved cognitive function in patients with PSCI, particularly in the domains of attention and executive function. They also observed reduced levels of amyloid‐β and altered functional connectivity in cognitive‐related brain networks following acupuncture intervention. L. Chen et al. (2016) similarly reported that acupuncture as an adjunct to conventional rehabilitation enhanced cognitive recovery in patients with acute ischemic stroke. The long history of acupuncture use in stroke management and its favorable safety profile support its potential role in PSCI treatment.

The modified Suanzaoren decoction, a traditional Chinese herbal formula, also showed promising effects in our analysis. Zhu et al. (2023) found that this herbal preparation effectively improved cognitive function and reduced comorbid insomnia symptoms in patients with PSCI. The cognitive benefits of Suanzaoren decoction may be related to its neuroprotective, anti‐inflammatory, and antioxidant properties, as well as its modulatory effects on neurotransmitter systems (Tai et al. 2020; Zhu et al. 2023). The study also reported reduced cortisol levels following treatment, suggesting that stress reduction may be one mechanism through which this intervention improves cognitive function. While herbal medicine represents a promising approach for PSCI management, more rigorous research is needed to establish its efficacy and safety profile.

TUS and moderate‐intensity aerobic exercise also demonstrated beneficial effects on cognitive function in PSCI patients. Wang et al. (2022) reported that TUS combined with cognitive rehabilitation improved cognitive performance, daily functioning, and neurophysiological parameters in patients with PSCI. The proposed mechanisms include enhanced cerebral blood flow, increased BDNF levels, and modulation of neural activity in cognitive‐related brain regions. Huang et al. (2024) found that moderate‐intensity aerobic exercise improved cognitive function in individuals with stroke‐induced mild cognitive impairment, particularly in the domains of executive function and processing speed. These findings align with a meta‐analysis by Oberlin et al. (2017), which demonstrated positive effects of physical activity on poststroke cognitive function.

In this study, although the comparison‐adjusted funnel plot showed that some points were slightly off‐symmetry, suggesting that there may be slight publication bias or small study effects, Egger's regression test results showed no statistical significance (*p* = 0.276), indicating that there was no significant systematic bias in the overall network. It is worth noting that when there are few intervention nodes in the network (seven nodes in this study) and the number of direct comparison studies is limited (most comparisons are only one to two studies), the visual judgment of funnel plots is easily affected by individual studies, while Egger's test has low statistical power due to the small sample size. Therefore, visual asymmetry does not necessarily mean that there is true publication bias. In addition, we examined the distribution of intervention branches and found no specific intervention (e.g., tDCS or Baduanjin) concentrated on the high‐effect side, suggesting that there was no over‐publication of a particular type of intervention.

Despite the encouraging findings, several limitations of this network meta‐analysis warrant consideration. First, the relatively small number of studies for some interventions limits the precision of effect estimates and the power to detect differences between treatments. Second, the heterogeneity in outcome measures, intervention protocols, and patient characteristics across studies introduces uncertainty in the comparability of results. While we attempted to account for this heterogeneity using SMDs and random‐effects models, some degree of clinical heterogeneity likely remains. Third, the follow‐up periods in most included studies were relatively short, limiting our ability to assess the long‐term effects of interventions. Fourth, most studies were conducted in China, which may limit the generalizability of findings to other populations and healthcare settings. Fifth, although there were large differences in the number of studies and sample sizes for different interventions in this study, the results of our random‐effects model and sensitivity analysis showed that this difference did not significantly bias the ranking calculation. However, future studies still need to further increase the number of direct comparison studies to improve the reliability and accuracy of network meta‐analysis. Finally, the quality of evidence in some included studies was only moderate, highlighting the need for more methodologically rigorous trials in this field.

These limitations notwithstanding, our findings have important implications for clinical practice and future research. The results suggest that non‐pharmacological interventions, particularly traditional Chinese exercises and neuromodulation techniques, may offer promising approaches for PSCI management. The favorable safety profiles of these interventions, coupled with their potential efficacy, make them attractive options for clinical implementation. However, treatment decisions should consider individual patient factors, including stroke characteristics, comorbidities, preferences, and access to different interventions.

While our analysis provides a broad ranking of interventions, clinical decisions should consider patient‐specific factors. For example, patients with mild PSCI with mobility retention may benefit most from active mind‐body exercises (such as Baduanjin) or moderate‐intensity aerobic training, which require cognitive and physical engagement. In contrast, individuals with moderate to severe cognitive or motor impairment may be better suited for noninvasive neuromodulation (e.g., tDCS) or acupuncture, which do not rely heavily on patient compliance. In addition, interventions such as modified sour jujube kernel decoction may be particularly suitable for patients with insomnia or anxiety disorders. Future trials should stratify participants based on baseline severity and functional status to generate evidence for personalized treatment algorithms.

Furthermore, the lack of consistent reporting of important clinical variables across studies, such as stroke type, time since stroke, intervention duration, and baseline cognitive impairment severity, prevented us from conducting meaningful moderator analyses. While all studies reported primary outcomes and quality scores, the heterogeneity in outcome measures and missing clinical details limit our ability to identify factors that may influence treatment response. Future studies should adopt standardized reporting of these key variables to enable more comprehensive meta‐analyses.

Future research should focus on conducting larger, methodologically rigorous RCTs with longer follow‐up periods to establish the sustained effects of interventions. Direct head‐to‐head comparisons between promising interventions would strengthen the evidence base and refine treatment recommendations. Additionally, studies investigating the optimal timing, duration, and intensity of interventions, as well as the potential benefits of combined treatment approaches, would provide valuable insights for clinical practice. Exploring the mechanisms underlying treatment effects through neuroimaging and biomarker studies would enhance our understanding of PSCI pathophysiology and inform the development of targeted interventions.

The integration of traditional Chinese medicine approaches with conventional rehabilitation strategies represents a particularly promising direction for future research. The cultural acceptability and widespread use of TCM interventions in many Asian countries, coupled with their emerging evidence base, suggest that these approaches may be valuable components of comprehensive PSCI management. Collaborative international research initiatives that bridge Eastern and Western medical paradigms could advance our understanding and treatment of PSCI.

In conclusion, this network meta‐analysis provides a comprehensive comparison of various interventions for poststroke cognitive impairment. The findings suggest that all evaluated interventions showed some degree of efficacy compared to cognitive training alone, with Baduanjin exercise and tDCS demonstrating the most promising results. These non‐pharmacological approaches offer potential benefits for cognitive function in stroke survivors and warrant further investigation in larger, more methodologically rigorous clinical trials. The integration of evidence‐based interventions from both conventional medicine and traditional Chinese medicine may provide a holistic approach to addressing the complex challenges of poststroke cognitive impairment.

## 5 Conclusion

PSCI represents a significant challenge in stroke rehabilitation, with profound implications for patients' functional recovery and quality of life. This network meta‐analysis synthesized evidence from 11 RCTs to compare the efficacy of various interventions for PSCI. The findings suggest that Baduanjin exercise and tDCS may be particularly effective in improving cognitive function following stroke, followed by acupuncture, modified Suanzaoren decoction, TUS, and moderate‐intensity aerobic exercise. These non‐pharmacological approaches offer promising alternatives or complements to conventional cognitive rehabilitation. However, the evidence base remains limited by the relatively small number of high‐quality studies and short follow‐up periods. Future research should focus on conducting larger, methodologically rigorous trials with longer follow‐up periods and direct comparisons between promising interventions. The integration of traditional Chinese medicine approaches with conventional rehabilitation strategies may offer a comprehensive approach to addressing the complex challenges of poststroke cognitive impairment.

## Author Contributions

H.L. and Z.Z. conceived of the study, P.Q. and J.W. participated in its design and data analysis and statistics, and P.X. and Y.Y. helped to draft the manuscript. All authors read and approved the final manuscript.

## Funding

The authors have nothing to report.

## Ethics Statement

The authors have nothing to report.

## Conflicts of Interest

The authors declare no conflicts of interest.

## Data Availability

All data generated or analyzed during this study are included in this article. Further enquiries can be directed to the corresponding author.
